# Controlled 3D co-culture of beta cells and endothelial cells in a micropatterned collagen sheet for reproducible construction of an improved pancreatic pseudo-tissue

**DOI:** 10.1063/5.0023873

**Published:** 2020-11-05

**Authors:** Haewon Seo, Jaejung Son, Je-Kyun Park

**Affiliations:** 1 Department of Bio and Brain Engineering, Korea Advanced Institute of Science and Technology (KAIST), 291 Daehak-ro, Yuseong-gu, Daejeon 34141, Republic of Korea; 2 KAIST Institute for Health Science and Technology, 291 Daehak-ro, Yuseong-gu, Daejeon 34141, Republic of Korea

## Abstract

The co-culture of beta cells and endothelial cells in constructing a pancreatic pseudo-tissue can provide a functional advancement for *in vitro* diabetic-related drug testing and biological studies or *in vivo* transplantation. In order to mimic the pancreatic tissue more similar to *in vivo*, it is necessary to control the microenvironment, including cell–cell and cell–extracellular matrix interactions. In this study, we report a geometrically controlled three-dimensional (3D) pancreatic model where MIN6 and MS1 cells are co-cultured within a micropatterned collagen sheet. In 4–10 days, depending on the cell seeding concentration, the MIN6 cells formed islet-like clusters surrounded by an endothelial MS1 cell monolayer. The MS1 cells also formed monolayers at the edge of the micropatterns connecting between the clusters, resulting in a blood vessel-like structure in which no cells were found. It was confirmed that the 3D co-culture structure was not formed in a non-patterned sheet and the structure also helped insulin secretion of MIN6 cells. By simply embedding the cell mixture and the hexagonal micropattern into the collagen sheet, we were also able to achieve the highly reproducible fabrication of a 3D pancreatic pseudo-tissue construct for *in vivo* and *in vitro* applications.

## INTRODUCTION

Diabetes is one of the diseases that cause most deaths around the world. It caused approximately 1.5 million deaths in 2012, and 2.2 million additional deaths were caused by high blood glucose levels.^1^ Moreover, the number of patients suffering from this disease has quadrupled since 1980, and today, more than 400 million people have diabetes. This is because insulin is not produced in a sufficient amount (type 1 diabetes, T1D) or the body uses insulin ineffectively (type 2 diabetes, T2D), leading to high glucose levels.

Glucose homeostasis is maintained by insulin, which is produced from the islets of Langerhans in the pancreas. The islets comprise several hormone-producing cells, but mainly insulin-producing beta cells (40%–70% depending on the species).^2^ Depending on the species, beta cells are clustered into spherical islets with a diameter of 50–200 *μ*m.^3,4^ The aggregation of beta cells is natural and shows an increased insulin secretion compared to single cells because the cell contact between beta cells allows synchronized calcium oscillations in response to glucose.^5,6^ Many studies for the *in vitro* formation of pseudo-islets have focused on the production of single-cell types, especially beta cells.^7–11^ However, an islet is a multi-culture of various cells and the interaction between them might not be fully understood. In this context, new findings can be gained by investigating such aspects.

Among the various cell types within the islets, endothelial cells (ECs) form blood vessels inside and outside the islets.^12–14^ As commonly known, blood vessels transport nutrients and gases to the cells within the islets. Thus, the *in vivo* transplantation of islets requires a preformed vascular network inside and outside the islets, which can readily connect to the recipient's blood vessels in a minimal amount of time. In addition to functioning as blood vessels, ECs also communicate with beta cells at the molecular level.^15,16^ The regulated secretion of the molecule by communication controls the level of cell survival, proliferation, insulin secretion, angiogenesis, and other gene expressions. Furthermore, since an impairment of beta cell functions involves such vascular dysfunctions,^15,16^ it is essential that an *in vitro* three-dimensional (3D) culture model of the pancreas consists of both beta cells and ECs.

Previous strategies on the construction of pancreatic 3D co-culture models include microwells^17–19^ and hydrogel scaffolds.^20–22^ The microwell-based islet formation is a relatively simple and easy method where single cells are mixed and loaded on the microwell, which has a non-adherent round bottom to enhance aggregation.^17–19^ These methods easily achieve homogeneity in size and roundness of the pseudo-islets in high-throughput yield, but they are limited in forming cell–extracellular matrix (ECM) interactions and handling the independent clusters for *in vitro* analysis or *in vivo* implantation. In the hydrogel scaffold methods, preformed pseudo-islets or extracted *in vivo* islets with endothelial cells are seeded on or embedded in a hydrogel scaffold.^20–22^ These methods result in tissue-unit constructions and, thus, require less laborious manipulation. Moreover, the hydrogels can offer cells with an *in vivo*-like environment, if the chosen hydrogels are similar to the ECM contents. However, these methods are not reproducible in size, position, and structure of the islets and endothelial networks. On the other hand, extrusion-based 3D bioprinting methods provide spatial patterning of the hydrogel, including islets and ECs via robotic automation.^23,24^ These methods can control the positions of islets and ECs homogeneously and reproducibly, but the printing resolution is limited to maintain the cell viability as well as the printing pressure and speed. Moreover, the printed hydrogel needs to maintain its shape during and after printing, which limits the hydrogel concentration. The high concentration of hydrogel cannot mimic an *in vivo*-like environment.

**FIG. 1.**
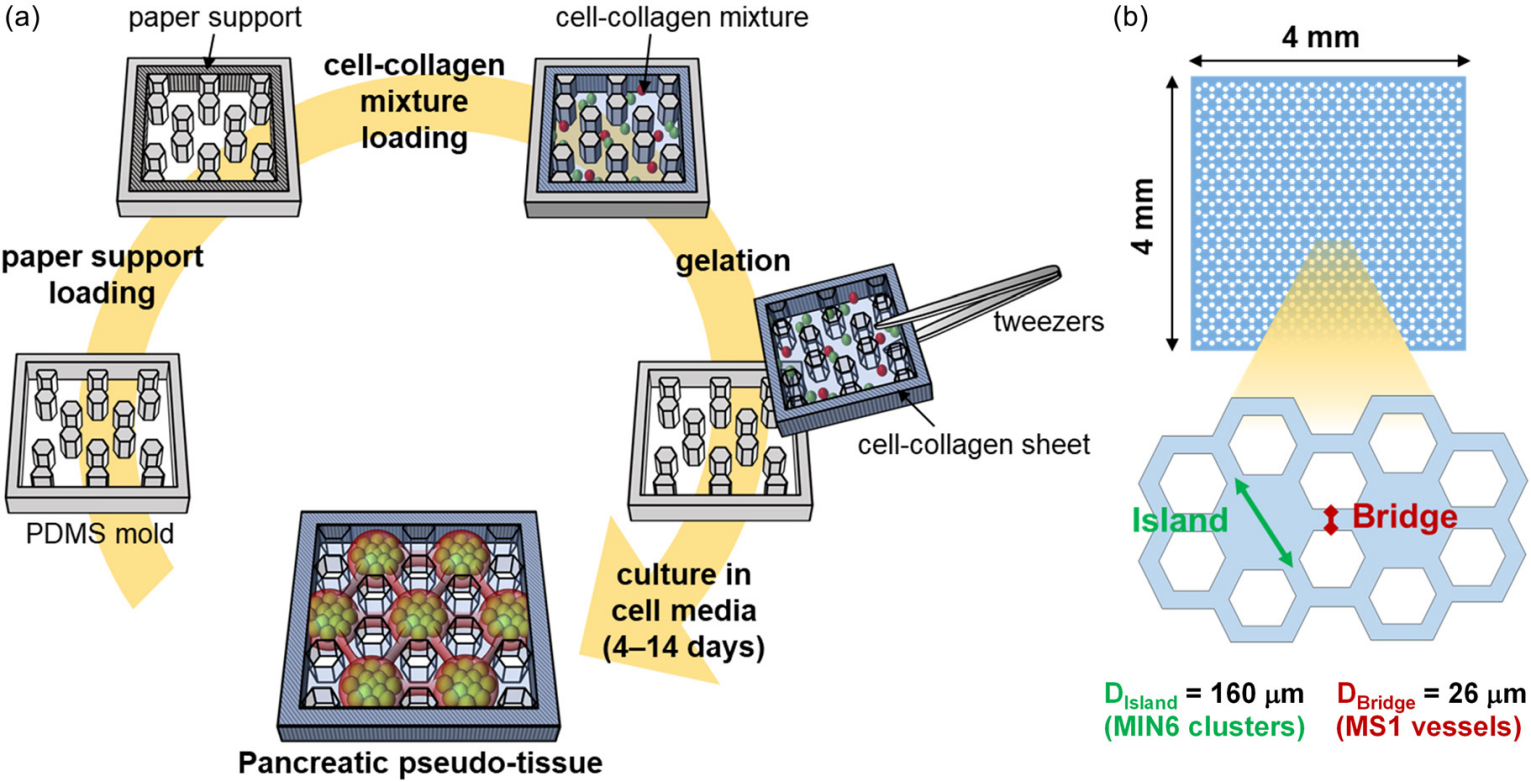
Cell-laden collagen sheet. (a) Schematic procedure of fabricating a micropatterned hydrogel sheet. (b) The dimension of the sheets. A hexagonal micropattern was chosen, where D_island_ is the diameter of the hexagon called an island and D_bridge_ is the width of the part connecting the two hexagons called a bridge.

Here, we propose a geometrically controlled pancreatic model constructed from a co-culture of beta cells and ECs (Fig. 1). With the following three strategies, we overcome the limitations of the previous co-culture approaches. First, pseudo-tissue fabrication is achieved at a high-throughput yield by a simple, easy seeding of the cell–hydrogel mixture. The cell seeding procedure involves a simple pipette mixing of beta cells and ECs with the collagen precursor and then an easy pipette loading of the mixture on the replica mold. Second, the reproducibility of islet formation within the pseudo-tissue is achieved by both the micropatterned sheet and the interaction with endothelial cells. In addition, the precise dimensions and shapes are homogeneous and controllable throughout the sheets and among the sheets. Third, an endothelial network surrounding beta cell clusters, which enables a pseudo-tissue-unit manipulation and analysis, is constructed by using a collagen sheet. Compared to our previous approach using the alginate sheet,^10^ the collagen sheet developed enables the 3D culture of ECs.^25^ It can also prevent the beta cells from escaping into a void between micropatterns so that the formed islets do not completely detach or unstably hang from the sheet. Therefore, the sheets can be conveniently handled, analyzed, and integrated with other devices.^26^

## RESULTS AND DISCUSSION

### Optimization of the co-culture condition in micropatterned sheets

First, the geometrically controlled pancreatic co-culture platform was fabricated using a micropatterned collagen sheet containing randomly mixed MIN6 (mouse pancreatic beta cell line) and MS1 cells (mouse pancreatic islet endothelial cell line) [Fig. 1(a)]. A paper support was combined with a collagen sheet for handling a freestanding collagen sheet. It was loaded into a micropatterned mold and was soaked with the cell-collagen mixture loaded subsequently. After the collagen has gelled at 37 °C, the collagen sheet can be harvested from the mold by picking up the paper support. The micropattern within the sheet was designed so that islands are connected by bridges [Fig. 1(b)].^10^ The island with D_island_ is expected to be occupied mainly by MIN6 cells and, thus, form an islet-like cluster within. The gap between two void hexagons is labeled D_bridge_ because this bridge is expected to be occupied mainly by MS1 cells and, thus, form a vessel-like structure. In this study, D_island_ was designed to be 140 *μ*m so that the resulting MIN6 clusters could achieve the biological islet diameter (100–200 *μ*m).^10^ D_bridge_ was designed to be 26 *μ*m so that the resulting MS1 vascular structure could achieve the biological vessel diameter (20–30 *μ*m).^2^

**FIG. 2.**
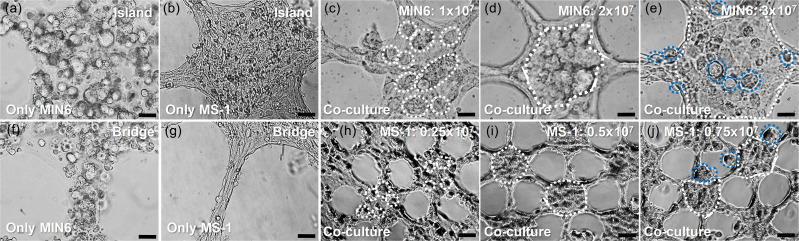
Mono-culture and co-culture of MIN6 cells and MS1 cells with collagen sheets. (a), (b), (f), and (g) Microscopic images of the mono-culture of MIN6 cells (a) and (f) and MS1 cells (b) and (g) on day 4. (c)–(e) and (h)–(j) Microscopic images of the co-culture of both MIN6 cells and MS1 cells on day 4. (c)–(e) Various cell concentrations of MIN6 cells with 1.0 × 10^7^ (c), 2.0 × 10^7^ (d), and 3.0 × 10^7^ (e) cells/ml at a fixed concentration of MS1 cells (0.5 × 10^7^ cells/ml). (h)–(j) Various cell concentrations of MS1 cells with 0.25 × 10^7^ (h), 0.5 × 10^7^ (i), and 0.75 × 10^7^ (j) cells/ml at a fixed concentration of MIN6 cells (2.0 × 10^7^ cells/ml). The white and blue dotted contours indicate the MIN6 clusters inside and outside the sheet pattern, respectively. Scale bars are 20 *μ*m (a)–(g) and 50 *μ*m (h)–(j).

To observe the morphological change of the sheet induced by each type of cell, MIN6 and MS1 cells were seeded to the collagen sheet and cultured for four days [Figs. 2(a), 2(b), 2(f), and 2(g)]. The concentration of the seeded cells was 1 × 10^7^ cells/ml for each sheet. In the MIN6 cell-laden sheets, the initial shape and dimension of the micropattern were maintained throughout the culture days [Figs. 2(a) and 2(f)]. The cells aggregated to form small clusters (less than 20 *μ*m) with heterogeneous sizes. This can be possibly explained by the aggregating characteristics of beta cells. As mentioned in the introduction, the interaction between beta cells increases the responses to nutrients.^5,6^ Some cells did not stay contained within the micropattern, but remained at the edge of the pattern or popped out into the void hexagon. This may be due to the increase in the cell number from cell proliferation. However, compared to alginate in the previous study,^10^ the cells hardly escaped out of the collagen. Although the hydrophilicity of alginate, which does not allow any binding of mammalian cell proteins, encourages cell escape, the hydrophobic binding sites of collagen are recognized by mammalian cells so that the cells can consume and interact with collagen fibers.^27,28^ Meanwhile, the sheets laden with MS1 cells resulted in a different morphological change from the sheets laden with MIN6 cells. In the MS1 cell-laden sheets, the pattern dimension was significantly changed from the initial spherical shape [Figs. 2(b) and 2(g)]. The bridge (D_bridge_) shrunk in half, and the diameter of the island also decreased. The pattern shrinkage seems to be due to an increased tension created from the MS1 cell elongation and the tight binding between the cells.^25^

To observe the effect of the cell concentration on sheet morphology, MIN6 cells from 1 × 10^7^ cells/ml to 3 × 10^7^ cells/ml and MS1 cells from 0.25 × 10^7^ cells/ml to 0.75 × 10^7^ cells/ml were seeded within the collagen sheet, which was then cultured until day 4. The concentration of MS1 cells was fixed at 0.5 × 10^7^ cells/ml when different MIN6 cell concentrations were compared [Figs. 2(c)–2(e)], and MIN6 cells were fixed at 2.0 × 10^7^ cells/ml when different MS1 cell concentrations were compared [Figs. 2(h)–2(j)]. MIN6 cells and MS1 cells at concentrations of 2.0 × 10^7^ cells/ml and 0.5 × 10^7^ cells/ml, respectively, resulted in a compact MIN6 cell cluster that filled the island surrounded by MS1 cells [see white dotted contours in Figs. 2(d) and 2(i)]. The MS1 cells also formed tight bridges that extend between the clusters. On the other hand, a lower concentration of MIN6 cells resulted in small-sized clusters [see white dotted contours in Fig. 2(c)], which were possibly insufficient to completely fill the island and to merge into a greater sized (100 *μ*m) cluster. At a higher concentration of MIN6 cells, the pattern dimension (D_bridge_ and D_island_) was larger [Fig. 2(e)]. This indicates that the higher MIN6 cell number occupied space, and thus, the MS1 cells were unable to surround the clusters and make the bridge so compact. At a lower concentration of MS1 cells, the MS1 cells were not fully aligned at the pattern edges, and the MIN6 cells formed small clusters [see white dotted contours in Fig. 2(h)]. At a higher concentration of MS1 cells, the width of the bridge (D_bridge_) decreased, but the middle-sized (50 *μ*m) MIN6 clusters were found in the void hexagons [see blue dotted contours in Fig. 2(j)]. A lower MS1 cell number fails to bring the MIN6 cells to the center to form a cluster, while a higher MS1 cell number fails to give sufficient space for the MIN6 cells to remain within the pattern. This suggests an effect of MS1 co-culture on increasing the MIN6 aggregation speed.

### Formation of co-cultured beta cell clusters over time

**FIG. 3.**
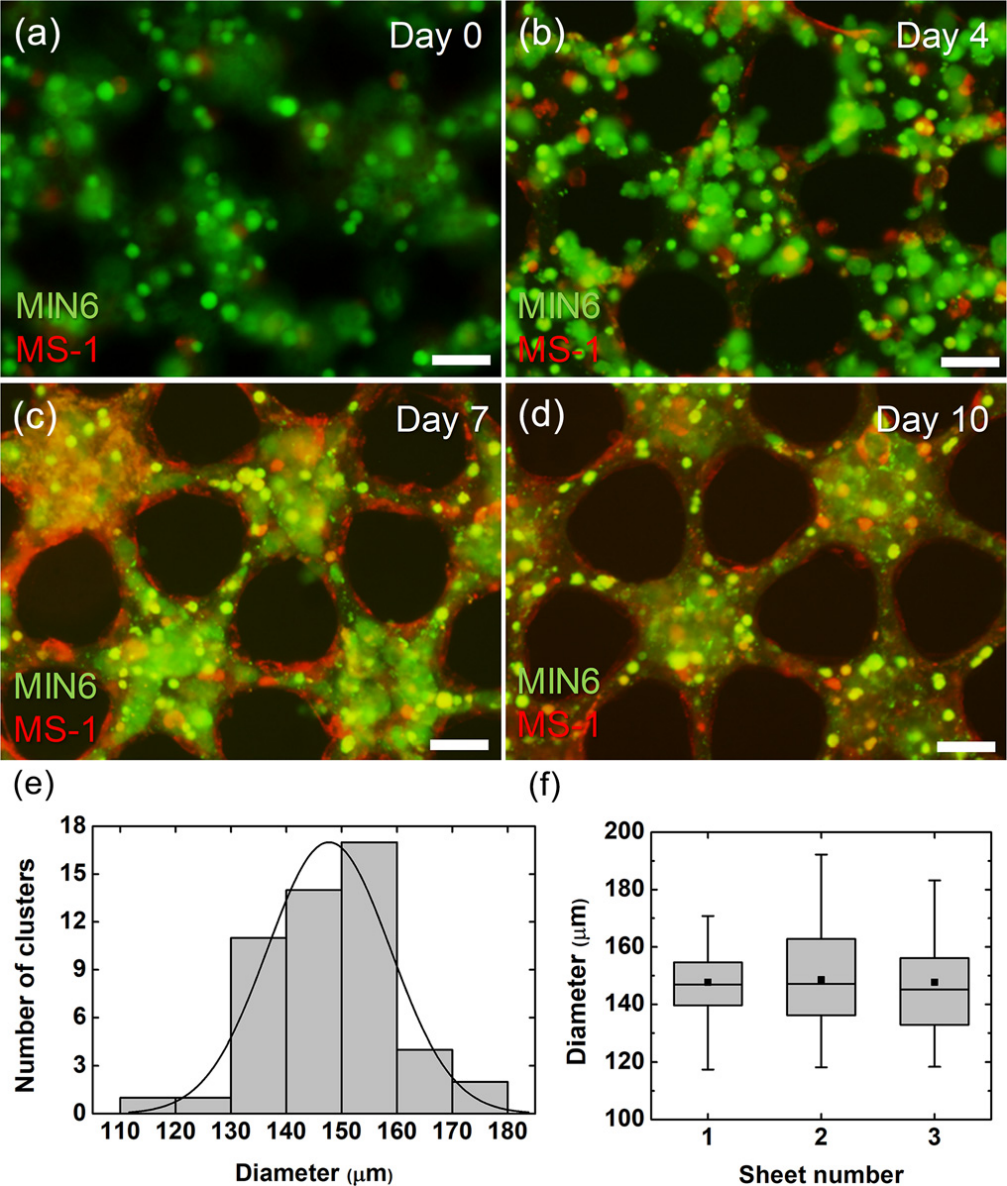
Formation of co-cultured beta cell clusters and the cluster characterization. (a)–(d) Fluorescence microscopic images of MIN6 and MS1 co-cultured collagen sheets on day 0 (a), day 4 (b), day 7 (c), and day 10 (d). MIN6 cells were stained in green, and MS1 cells were stained in red. (e) Distribution of the cluster diameter throughout the sheet. (f) Comparison of cluster diameters among the three sheets (*n* = 40). Scale bars are 50 *μ*m.

Figure 3 shows the fluorescence images of the co-cultured sheets on days 0, 4, 7, and 10 to observe the morphological changes of the co-cultured cells within the sheet. On day 0, the cells are randomly distributed among the micropattern [Fig. 3(a)]. It can be seen that space is mostly occupied by MIN6 cells (shown in green), due to a 10-fold difference in the cell number compared to the MS1 cells (shown in red). On day 4, it can be seen that the dimension of the micropattern (D_island_ and D_bridge_) has slightly shrunk [Fig. 3(b)]. Similar to what happened in mono-culture [Figs. 2(a) and 2(f)], the MIN6 cells aggregated to form small clusters (less than 20 *μ*m). On the other hand, the MS1 cells seemed to have proliferated and migrated mainly to the pattern edge, although some were still distributed within the pattern center. Some MS1 cells were elongated, but not as much as in mono-culture [Figs. 2(b) and 2(g)] due to the lower cell number. On day 7, the MS1 cells have proliferated at the pattern edge to form a vessel-like structure that encloses the pattern, while the small MIN6 cell clusters have aggregated to form bigger clumps [Fig. 3(c)]. On day 10, the MIN6 cells have formed a compactly packed cluster that fills the island, while the MS1 cells tightly surround the MIN6 cell clusters [Fig. 3(d)]. The width of the bridge (D_bridge_) has decreased even more, which may be due to tighter binding between the MS1 cells. A decreased island diameter can also be explained by the difference in green fluorescence intensity between the bridge and the island, indicating that MIN6 cells may have migrated from the surrounding MS1 cells into the center of the island.

Furthermore, the diameter of the formed MIN6 cell clusters was analyzed to assure the homogeneity of the cluster sizes throughout the sheets and among the different sheets [Figs. 3(e) and 3(f)]. The diameter distribution in one sheet shows that most clusters are formed between 130 and 160 *μ*m size, with an average of 148 *μ*m [Fig. 3(e)]. The distribution among the three sheets also shows that the cluster sizes are homogeneous between 100 and 200 *μ*m, which is similar to the biological pancreas [Fig. 3(f)]. The overall homogeneity in diameter shows that the pseudo-tissue with clusters embedded could be produced reproducibly.

### Morphology of co-cultured cells within the micropatterns

**FIG. 4.**
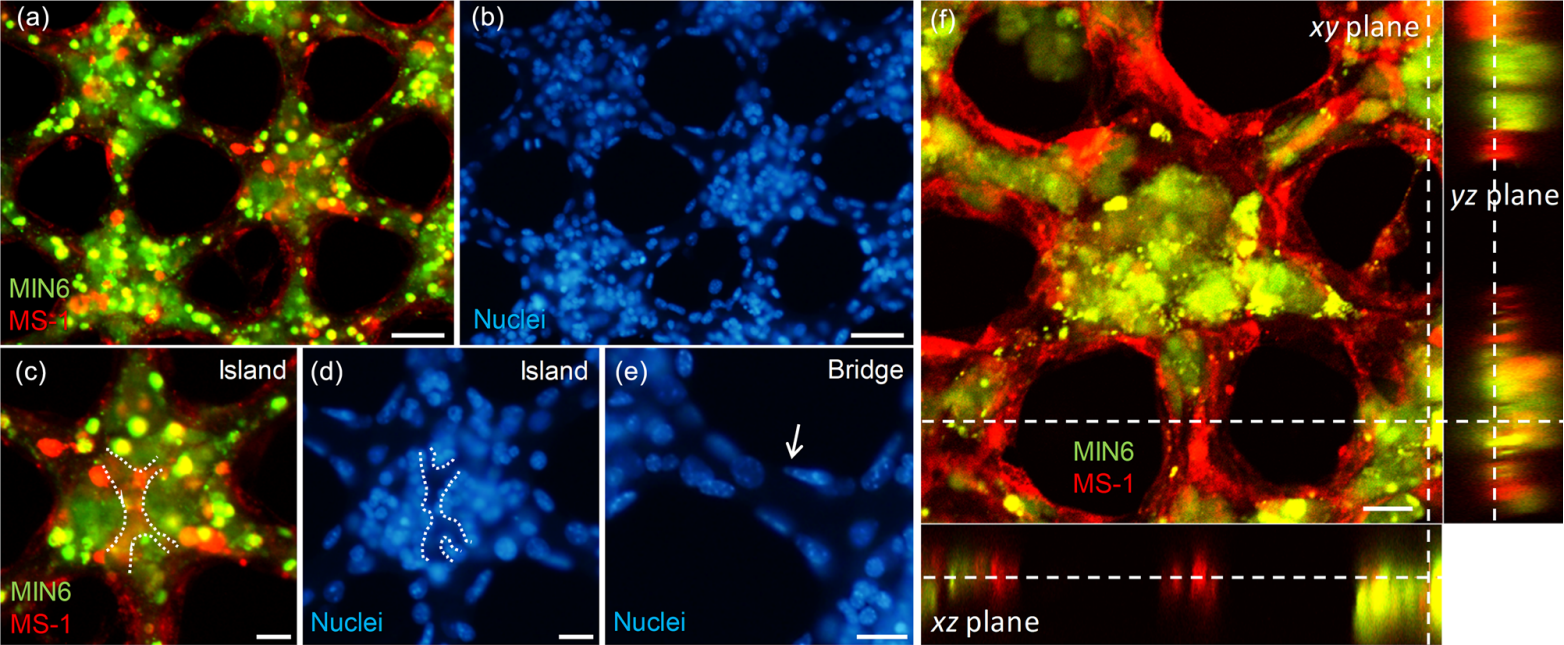
Fluorescence microscopic images of co-cultured beta cell clusters within collagen sheets. (a) A sheet with MIN6 cells stained in green and MS1 cells stained in red on day 7. (b) The same sheet containing both cells with the nuclei stained in blue. (c) and (d) Expanded images of (a) and (b) at the island area. The white dotted lines indicate the edge of the MS1 cells within the cluster. (e) Expanded image of (b) at the bridge area. The white arrow indicates MS1 cells present in the edge of the bridge pattern, not in the interior of that. (f) Sectioned images of co-cultured beta cell clusters on day 4 in the *xy* (center), *xz* (bottom), and *yz* (right) plane obtained from confocal imaging. The white dashed lines indicate the same position of the collagen sheet. Scale bars were 50 *μ*m (a) and (b), 20 *μ*m (c)–(e), and 25 *μ*m (f).

To check the cell arrangement within the sheet, the cell nuclei were stained in blue out of the sheet with green-labeled MIN6 and red-labeled MS1 cells [Figs. 4(a) and 4(b)]. After cell seeding, the harvest of cell-containing collagen sheets combined with a paper support showed a success rate of approximately 60% from a mold, which can be increased by changing with the mold coating method.^25^ However, after harvest, the handling of the sheets is always successful without tearing, so that it is possible to perform nuclear staining after cell culture. In the bridge, a monolayer of cells is lined on the edge, while no cells are observed inside [Figs. 4(b) and 4(e)]. This indicates that the MS1 cells form a monolayer of a vessel-like structure. A vessel-like structure was seen over the island, where the majority of MIN6 cells were gathered into a cluster [see the white dotted line in Fig. 4(c)]. When we observed the same position in the nuclei-stained image, we noticed that the vessel-like structure was hollow inside, as no nuclei were seen [see the white dotted line in Fig. 4(d)].

In addition, a confocal image was taken from a sheet with MIN6 and MS1 cells to observe the morphology in the *z*-axis [Fig. 4(f)]. The MIN6 clusters do not exactly match the spherical shape of the pancreatic islet, but rather flat. On the other hand, the width and height of the vessel-like bridge were within 20 *μ*m with an aspect ratio close to 1:1. Based on the thickness differences between the MIN6 cluster in the island and the MS1 endothelial bridge, the flat shape seems to result from the tension caused by the MS1 cells surrounding the clusters. The roundness may be achieved by controlling the cell seeding concentration or the height of the sheet depending on the height of the mold and the paper support.

### Cell viability change over time

**FIG. 5.**
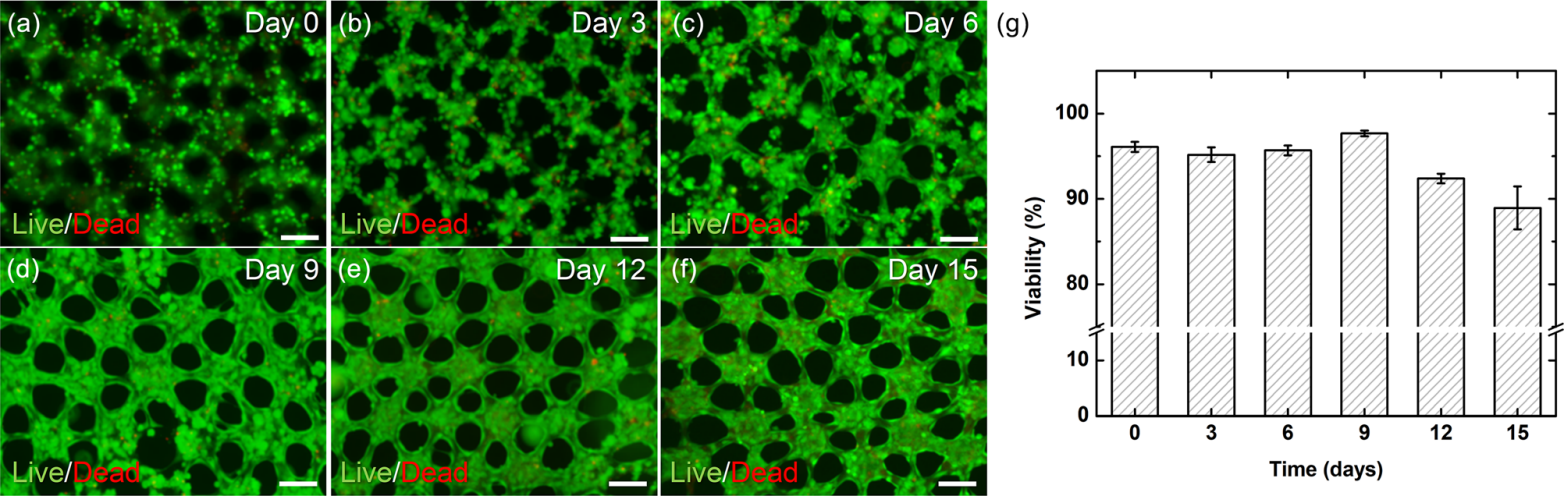
Cell viability of pancreatic co-cultured cells within collagen sheets. (a)–(f) Fluorescence microscopic image of cells with live/dead staining on day 0 (a), day 3 (b), day 6 (c), day 9 (d), day 12 (e), and day 15 (f). (g) A graph of viability changes over 15 days (*n* = 3). Scale bars are 100 *μ*m.

For a prolonged observation of cellular changes *in vitro* or for *in vivo* transplantation, the cells within the sheet need to be maintained in high viability. Cell viability was maintained more than 95% until day 9 but then decreased by day 15 (Fig. 5). The culture medium was replaced every three days right before the images were taken, and thus, the dead cells moving out of the sheets may have been washed away and not counted in these data. Before the vascular network was completely formed (day 0–9), dead cells could escape out of the sheet and be removed during the medium change. Once the endothelial network is formed (between day 9 and 12), dead cells cannot escape out of the sheet. It seems that the ECs are tightly bound surrounding the collagen micropatterns and the resulting vessel-like structures are impermeable to the dead cells. Nonetheless, the cells co-cultured in the patterned collagen sheets maintained a high cell viability of over 85% for about two weeks.

### Effect of micropatterns for co-cultured beta cell clusters

**FIG. 6.**
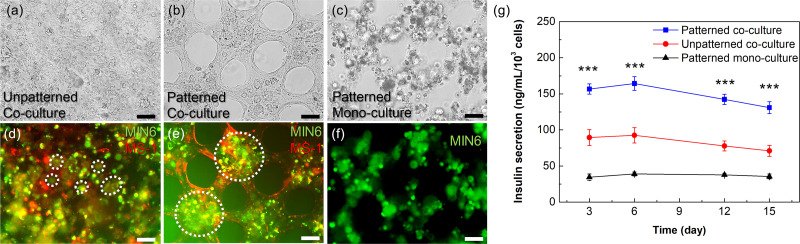
Effect of micropatterns for co-cultured beta cell clusters. (a), (b), (d), and (e) Microscopic images of MIN6 and MS1 co-cultured sheets without (a) and (d) and with (b) and (e) micropatterns. (c) and (f) Microscopic images of a MIN6 mono-cultured sheet with micropatterns. (a)–(c) Bright images and (d)–(f) fluorescence images taken on day 12. MIN6 cells are stained in green, and MS1 cells are stained in red. The white dashed circles in panels d and e indicate smaller clusters (approximately 20–50 *μ*m) and larger clusters (greater than 100 *μ*m), respectively. (g) A graph of the insulin secretion level over time in MIN6 cells mono- and co-cultured in micropatterned sheets and the cells co-cultured in bulk sheets without micropatterns (*n* = 4). Significant differences between the three groups were determined using a one-way ANOVA followed by Tukey's post-hoc test. ^***^p-value < 0.001 (vs patterned co-culture group). Scale bars are 50 *μ*m.

To observe the effect of having the micropattern within the co-cultured sheet, a bulk collagen sheet without a micropattern was compared (Fig. 6). Note that the hexagonal micropattern gives three effects. First, the area is reduced to 62.5% of the sheet without the pattern, which means that the cells can occupy less space. Second, the shape of the micropattern gives the cells a guideline to grow along. Finally, nutrients and gas can reach the cells more effectively, because the culture medium can flow through the cavities of the micropattern.

Until day 12, the cells within the micropatterned sheets aggregated into each island, forming a cluster with a diameter greater than 100 *μ*m [Figs. 6(b) and 6(e)]. On the other hand, the MIN6 clusters within the bulk sheet were randomly distributed and were limited to a smaller and non-homogeneous diameter around 20 *μ*m [Figs. 6(a) and 6(d)]. There were also morphologic changes in MS1 cells within the micropatterned sheets compared to bulk sheets. The MS1 cells were lined along the edge of the pattern in a thin and elongated shape [Fig. 6(e)], while the cells maintain a non-elongated but rather circular shape in the bulk sheets [Fig. 6(d)]. Mono-cultured MIN6 cells within the micropatterned sheet showed more controlled distribution compared to those within co-cultured bulk sheets [Figs. 6(c) and 6(f)]. However, they did not tightly aggregate without MS1 cells.

These results were also associated with differences in the function of insulin secretion. The MIN6 insulin secretion levels of micropatterned sheets were significantly about 1.5 times higher than those of bulk sheets [Fig. 6(g)]. In addition, the secretion levels of the co-cultured conditions were significantly 2–3 times higher than those of the mono-cultured condition. The micropattern improved the pancreatic function further although communication between beta cells and ECs promoted the function as previously known. This is because the tendency of vascular cells to grow along the edge of the micropattern promoted the aggregation of beta cells. If the induced pluripotent stem cell-derived pancreatic cells are used for future research, we believe that our model can be used to construct an improved pancreatic pseudo-tissue model for *in vivo* purposes such as cell replacement therapy.

## CONCLUSIONS

Here, we constructed a geometrically controlled pancreatic pseudo-tissue model where MIN6 and MS1 cells were co-cultured using freestanding micropatterned collagen sheets. In 4–10 days, depending on the cell seeding concentration, MIN6 cells formed islet-like clusters surrounded by an MS1 cell monolayer. The MS1 cells also formed monolayers at the edge of the hexagonal micropattern, resulting in a blood vessel-like structure with no cells found inside. MS1 intra-islet vessels were formed, but they had a greater diameter than the *in vivo* intra-islet capillaries. We were able to achieve a high-throughput yield in pseudo-tissue fabrication, by simple, easy seeding of the cell mixture. The high reproducibility of the islet and the endothelial network was also achieved by the hexagonal micropattern, which was proved by the cluster size homogeneity throughout and among the sheets. We were able to create a pancreatic pseudo-tissue model with improved functionality, by using the micropatterned collagen construct for enhanced cell–cell interactions within the matrix. It is expected that the reproducible and easily handled pancreatic co-culture model can be used for *in vivo* implantation, which enables fast anastomosis with host vasculatures, or for *in vitro* diabetic drug testing with more physiologically relevant microenvironments. As one of the examples of *in vitro* applications, our pseudo-tissue model can be applied to an integrated microfluidic device for a simple assay based on a drug concentration gradient. By integrating a pancreatic cell sheet into a microchannel,^26^ it can be used for biological studies or drug testing by investigating the difference in viability and expression of pancreatic cells with respect to different drug concentrations.

## METHODS

There are no experiments on human or animals in this study; therefore, ethics approval is not required.

### Fabrication of sheet mold

Cell-laden collagen sheets were fabricated using poly(dimethylsiloxane) (PDMS) molds. These micropatterned PDMS replica molds were prepared through traditional photolithography and soft lithography. SU-8 2100 (MicroChem Corp, Westborough, MA, USA) was poured on a bare silicon wafer and was spin-coated to achieve a thickness of 220 *μ*m. It was then exposed to ultraviolet (UV) light under a photomask that was printed with the desired micropattern. The unexposed and, thus, uncured part of the photoresist (PR) was removed by sonicating in a SU-8 developer. The PDMS prepolymer was mixed with a curing agent (Sylgard 184 silicone elastomer kit; Dow Corning, Midland, MI, USA) at the ratio of 5:1 and was degassed in a vacuum chamber. The PDMS mixture was poured on the SU-8 master mold, which was coated with a polyurethane resin mold release agent (Flex-A; Nabakem, Pyeongtaek, Korea) to achieve easier detachment of PDMS. The PDMS was cured at 120 °C for 15 min.

Since the resulting sheet molds were to come into contact with cells in the following experiments, additional steps were required to achieve sterile conditions. To remove the residual polyurethane release agent, the PDMS replica molds were each sonicated for 30 min in ethanol and de-ionized distilled water. The molds were then autoclaved at 120 °C for 15 min and dried at 65 °C overnight. The molds were exposed to UV light for 30 min before being used in a cell experiment.

### Fabrication of a cell-laden collagen sheet

The cell-laden collagen sheet was fabricated as described in Fig. 1(a). Collagen (Collagen type I, rat tail, 3–4 mg/ml; Corning, NY, USA) was chosen as a hydrogel material instead of alginate as in our previous studies to increase the attachment of the mammalian cells to the hydrogel fibers and, thus, provide an environment similar to the biological ECM. To improve the manual handling of collagen, a square donut-shaped paper support (outer side length: 7 mm, inner side length: 4 mm) was integrated on the periphery of a sheet. Filter paper (Whatman qualitative filter paper, grade 4, 54, 154; GE Whatman, Maidstone, Kent, UK) was cut into the corresponding shape by a laser cutter. The paper was sterilized through three rounds of sonication for 30 min in acetone and ethanol. For additional sterilization before use, the paper supports were autoclaved at 120 °C for 15 min, dried overnight in a 65 °C oven, and exposed for 30 min under UV light. The hydrogel precursor was prepared by mixing collagen with 1 M NaOH and 10× phosphate-buffered saline (PBS) according to the manufacturer's protocols. The MIN6 cells and MS1 cells were prepared through trypsin-EDTA treatment and centrifugation.

MIN6 cells were generously donated by Professor Hail Kim, Graduate School of Medical Science and Engineering, KAIST (Daejeon, Korea), and MS1 cells were obtained from the American Type Culture Collection (ATCC CRL-2279; Manassas, VA, USA). Since MIN6 cells are known to vary in the characteristics depending on the passage number, 30–40 passaged cells were mainly used in this study.^29^ The prepared cells were mixed with the hydrogel precursor so that the desired cell concentrations were achieved between 1.5 × 10^7^ cells/ml and 3.5 × 10^7^ cells/ml in total. Mixing was done simply by pipetting. The surface of the PDMS mold was temporarily gained hydrophilicity via a one-minute plasma treatment so that 10–15 *μ*l cell–hydrogel precursor mixture was pipetted on the mold and spread on the PDMS surface to form a thin layer, without capturing a bubble between the micropatterns. The precursor mixture on the mold was crosslinked within a 37 °C cell incubator for 30 min. The gelated sheets were detached from the mold by easily grabbing the connected paper support with the tweezers.

### Cell culture within the collagen sheet

Each sheet was placed in a 4-well cell culture plate (SPL Life Sciences, Pocheon, Korea) filled with 850 *μ*l of cell culture medium. Since the sheet contained two different cell types, the medium was prepared by mixing a 1:1 ratio of the typical culture medium used to culture each cell type, resulting in the following composition: Dulbecco's modified Eagle's medium with 4.5 g/l glucose supplemented with 11.3% fetal bovine serum, 100 mg/l penicillin–streptomycin, and 17.9 *μ*M 2-mercaptoethanol. The cells were cultured in a cell incubator (37 °C, 5% CO_2_, 95% humidity) up to 15 days so that the cells could form a tissue-like structure within the sheet. The culture medium was replaced every three days.

### Analysis of cell morphology, viability, and function

To analyze the cell morphology, MIN6 cells were stained with 10 *μ*M Celltracker green CMFDA (Invitrogen, Carlsbad, CA, USA) and MS1 cells were stained with 10 *μ*M Celltracker red CMTPX (Invitrogen). The nuclei of both cells were stained blue with Hoechst 33258 (Thermo Fisher Scientific, Waltham, MA, USA). The morphology change of each cell type was observed every three days using a fluorescence microscope (IX52; Olympus, Tokyo, Japan). Morphology in the *z*-axis was observed using a confocal microscope and was recorded using image processing software (NIS-Elements; Nikon Instruments Inc., Melville, NY, USA). The reconstruction of a 3D image taken from a confocal microscope (Eclipse Ti; Nikon Instruments Inc.) was achieved using the software from sequential 1 *μ*m slices in the *z*-axis. The occupying area, the perimeter, and the Feret diameter of the MIN6 clusters were obtained using image processing software (Image J: http://rsbweb.nih.gov).

To observe the viability, the cells were live/dead stained with 10 *μ*M of calcein AM (green) and 4 *μ*M of ethidium homodimer-1 (red). The percentage of live cells within the sheets was obtained by calculating the ratio of live cell pixels to total pixels of live and dead cells from the fluorescence images. To quantitatively analyze the functionality of MIN6, insulin secretion was measured through a rat/mouse insulin enzyme-linked immunosorbent assay (ELISA) kit (Millipore, Burlington, MA, USA). The culture medium replaced every three days was collected to obtain secreted insulin. Before performing the insulin ELISA, the collected culture medium was diluted 5000-fold.

### Statistical analysis

Statistical analysis of data is expressed as the means ± standard deviation. The *p*-values were calculated using one-way ANOVA with Tukey's post-hoc test. The statistical significance threshold was set at ^***^p < 0.001. The sample sizes are indicated in the figure captions.

## AUTHORS' CONTRIBUTIONS

H.S. and J.S. contributed equally to this work.

## Data Availability

The data that support the findings of this study are available from the corresponding author upon reasonable request.
